# Diseases of Colliery Operatives, and Their Preventible Causes

**Published:** 1857-04

**Authors:** William I. Cox

**Affiliations:** Corresponding Member of the Epidemiological Society of London.


### ON THE DISEASES OF COLLIERY OPERATIVES; AND THEIR PREVENTIBLE CAUSES.

By WILLIAM I. COX, M.R.C.S., etc.; Corresponding Member of the Epidemiological Society of London.

(*Concluded from* vol. ii, p. 378.)

Having now considered the chief sources of the prevailing physical ailments of colliery operatives, I will briefly conclude this imperfect essay with suggesting a few palliative or remedial measures and aids towards the amelioration of the bodily, and also of the moral condition of this class of artizans.

1. *Baths and Wash-houses.*—"Cleanliness is next to godliness." Corporeal and spiritual uncleanness are in strong affinity and close relationship with each other. It is nearly impossible to over estimate the value of thorough and regular

---

ablution.* To dwell on the advantages of a healthy condition of the skin, and a free, unimpeded performance of its functions, would be, on this occasion, quite unnecessary; but the beneficial results of preserving the cutaneous surface in a normal state, are by no means limited to the maintenance of physical health, to the warding off disease. A human being, living constantly in dirt and filth, never knows self-respect; or, at most, seldom retains its inestimable possession for any long period of time. Rarely, indeed, has such an one the "*os sublime*" to lift erect towards his Maker, proudly and nobly conscious of the heaven-bestowed faculties of reason and intelligence: he feels cowed and debased in the company of his cleanlier and more decent fellow men: he feels the existence of a barrier between himself and them, arising as much from the obvious difference in externals, as in refinement of speech or cultivation of mind. Hence he shuns, rather than seeks, the society of his betters on all occasions, and the unhappy feeling of class distinction thus becomes inveterate amongst colliers (indeed, is far stronger in them than in other artizans) and is readily transmitted from generation to generation, until it becomes apparently in-born.

1. *The establishment of baths and wash-houses* in the immediate neighbourhood of the coal pits, would unquestionably do much to obviate the evils I have alluded to. Their moral influence would not be confined to this. The lax morality and contemptuous indifference towards the maintenance of sexual modesty, which I have spoken of in my first paper as peculiarly characterising both men and women in the coal districts, is partly attributable to the odious and common practice of the youths of both sexes stripping on their return home, and performing their apologies for ablution almost in a state of nudity, in the same apartment, and in open and undisguised view, not only of parents, but of unmarried sisters and brothers, and of adult lodgers. I have frequently been a disgusted witness to these gross and indecent spectacles. I have seen a young unmarried female stripped nearly to the hips, washing herself in the common sitting-room (*house-place*, as it is termed), which contained two full grown male lodgers. I have also frequently seen men similarly exposed in the presence of sisters and female strangers.

The premises necessary could be built and carried on at a very small expense to the proprietors of collieries, and the

* Colliers and their families have in general a horror of water. "Eh, doctor," said a collier's wife on a recent occasion to the writer, "sure, it 's *good wholesome dirt* (!) He 'd be getten the rheumatics if he washed all over; and so often as your 're wishing, 'tant gradely for coalers, I tell 'ee."

pecuniary cost would be amply compensated in the improved moral and physical status of the workpeople. In most *plants* there is generally one shaft needing continual pumping, and the water so raised (and which is now deemed a troublesome nuisance, difficult to be got rid of) might be readily turned into a reservoir, and after, perhaps, some necessary cheap filtering, be heated for the bath at a trifling cost.

The programme of the operative's day may be as follows :— In the morning, having reached the works, he enters a room adjoining the bath, puts on his pit dress—leaving his house garments in its place : he then proceeds to the scene of his labours. On coming up from the pit at the conclusion of his day's work, he repairs to the building, divests himself of his pit clothes, plunges into the bath ; and, after a brief but thorough ablution in the water (which in the summer may be at the common temperature, but should be heated during the winter to 90°), dresses himself once more in the ordinary clothes which he put off in the morning on his arrival. Now, by this arrangement, the operative (*a*) is secured a free and healthy action of the skin, as far as that *can* be done by external measures ; and (*b*) he is not compelled to appear in the streets, in his way to and from work, in his rude and unseemly pit habiliments. If it be objected that this programme involves a certain amount of trouble in changing clothes, etc., which by the workmen may be deemed irksome and unnecessary, such an alleged obstacle to its adoption will disappear on examination ; for it is *now* the almost universal custom amongst them to throw off the cumbersome pit dress, and put on their ordinary garments on returning home.*

2. *Improved Dwellings.*—A vast improvement in the social habits and physical welfare of the operatives would, doubtless, be effected by their provision with healthy, well ventilated dwellings, and a cheap, thorough water supply where this great desideratum is deficient. It is much to be regretted that the system at work in the Welsh and Monmouthshire coal fields (the colliers being there resident in cottages built by the masters in the immediate neighbourhood of the works) is not generally adopted in Lancashire. The operatives here live wherever they can find what they deem suitable residences. Vain are nearly all the attempts to better the condition of a class, whilst their homes are so grossly neglected. It is the error of many benevolent and well-intentioned philanthropists of the present

* The author will be happy to forward (on application) a small sketch or plan of the proposed building. He could not, of course, encroach on the space of this Journal by its introduction.

day to suppose that the self-denying virtues of temperance, of sobriety, of chastity, can be planted with success in the habitations of wretchedness, and their flourishing and permanent growth secured by oaths and vows. A sad mistake! In dwellings offensive to every sense, and among human beings embruted by constant intemperance, and surrounded by every species of bodily uncleanness, "no salutary plant takes root;" or if, indeed, obtaining a transient hold, speedily withers in a soil so adverse, in an atmosphere so ungenial. And here let me remark, that no one at a distance, and personally unacquainted with the actual condition and requirements of the poorer classes, can be possibly in a position to judge correctly on these important questions. However unpalatable the task, one must mix with and observe, and that with unprejudiced vision, ere one can venture to indicate the real sources of evil, and suggest preventives. The medical man, the general practitioner in humble life in many an obscure village or filthy haunt of the bustling city, becomes (provided he be ordinarily gifted and worthy his calling) a fitter judge of the remediable evils affecting the lives of the people, than probably one-half the members of both Houses. It is ever thus in the search for knowledge of true practicable value. Would anyone pick up precious truths? He must bend down and look for them—it may be even in the mire and slough of human wickedness and woe. Everywhere the "pearl of great price" is hidden and embedded in a shell, which has no form or comeliness; and knowledge of this kind, likely to benefit the future, is cumulative, of slow growth, piled up by little and little, won by untiring perseverance, by patient labour.

3. *Abolition of Female Labour.*—The abomination of female *employées* at the mouth (or brow, as it is called) of the shafts, wearing the male garb, should be swept away. I have already spoken of the utter demoralisation and destruction of all self-respect and sense of female shame, that *directly* results from this revolting usage; and the evil effects are, of course, not limited to the persons of the female operatives. The bodily welfare and moral *status* of their husbands (or of the men with whom they live in concubinage), are thereby indirectly lowered and deteriorated. These women sink into a condition of moral hermaphroditism—partaking of the mental characteristics of both sexes, in the coarser, baser, and more corrupt manifestations, but destitute of the virtues that should adorn either. The state of mind and morals reacts on the physical condition, and thus their unhappy offspring necessarily suffer from their degeneracy. Until a welcome Act of Parliament shall for ever forbid the employment of females (in any garb) at, or near, coal pits; a powerful check might be given to the maintenance of the system by the masters refusing their labour, as far as possible, and in other ways throwing the utmost discouragement on a custom so much "more honoured in the breach than in the observance." Moral suasion in this, as in other matters, would do much, if steadily enforced.

4. *Schools, Classes, and Lectures.*—It is much to be feared that but little can be anticipated in the way of elevating the mental condition of colliers and miners, until legislative enactments shall lay the foundation (as indubitably they ought to have done long since) of a simple, but permanent educational plan. We have been, indeed, long awaiting, and in vain, the advent of some Government measure, some scheme of tuition, which might happily disperse the Cimmerian gloom of popular ignorance and depravity which broods, *par excellence,* over the coal fields of Northern England. The question, "when will it come?" is yet unanswered. How long are we to remain indignant in *statu quo,* whilst waspish senators bandy personal sarcasms in the chambers of the legislature; whilst youthful lordlings exercise their polished weapons of wit and *repartie* upon some idle question; whilst pompous declamators, enraptured with the charms of their own vapid eloquence, dilate at weary length on the "visionary schemes" of the unflinching and earnest advocates of peace, sobriety, and sanitary reform; whilst frothy sophists sneer at education for the masses, and hold up beer—that curse of the labourer of every class—to universal admiration as "liquid bread"? Meanwhile, we live in a moral chaos. Respectable citizens daily run the hazard of *garottement* in the public streets! The unfortunate artizan lives, breeds, and rots in a filty hovel; his offspring, with no knowledge of God or respect for man, make rapid and unheeded strides to the possession of their dark inheritance of woe and crime, through an unnatural childhood, passed in a home revolting to every well-trained sense; the germs of every zymotic poison grow strong, and multiply in the atmosphere of the hideous den, the squalid court, the foul and reeking sewer. And, worst of all still, the untended precocious bud of youthful passion is, in both sexes, forced and blighted into ghastly shapes, in the hot polluting air, stagnant in homes of physical and moral corruption. But this is by-the-bye. *Revenons à nos moutons.* Why, I may ask, have we not, at all events, a bill rendering the school education of the children of colliery operatives compulsory at a certain age, as it is already with respect to the offspring of factory spinners and weavers? The alleged interference with natural rights of parents and guardians is not greater in the one case than in the other. My own experience in the peculiarities of temper and character in colliers (at least, in Lancashire) has convinced me that it is now nearly hopeless to leave the training of the rising generation in the hands of their natural protectors. The parents themselves, having received no education, are prejudiced against it. They have no faith in its advantages, and, generally speaking, despise it, and will not have their children taught or instructed by others if they can avoid it.

Yet, in the mean time, much may be hoped from the establishment of small schools by the masters, in the near vicinity of the operatives' cottages, for the children of the workmen, and more especially by forming evening classes or adult schools (with the delivery of lectures) for the operatives themselves. The advantage derivable from this last-named measure being, that the parents learning by their own experience of its advantages to estimate, however feebly, the blessings of knowledge, discard their former prejudices, and become desirous to secure for their children similar benefits. The truth of this assertion, and a good illustration of the success attending persevering effort (if but slight and partial) towards the amelioration of the collier's social condition, has been afforded in my own neighbourhood. A highly respectable firm of coal owners, the active and managing partner of whom is a gentleman much impressed with the moral deficiencies of the *employés*, and strongly desirous of their permanent benefit, established, recently, a day school for children, which for some time struggled on with but small results that could be deemed satisfactory. Then an evening lecture was set on foot, to be delivered weekly; the incumbent of the district and his zealous and excellent curate kindly undertaking the duty alternately. Now, soon after the commencement of these lectures, the attendance at the children's school was observed to improve, and the numbers to increase, and up to the present time *continues* to improve. The school instruction, however, should not be a free gift. A small charge should be made for each child; for these people, with a species of pride somewhat peculiar, and conscious of being really able to pay, cherish a kind of disdain towards all things gratuitous, every absolute *bonus*. It is found that lectures with free admission rarely succeed in the coal districts; but entertainments of that class, necessitating the outlay of two-pence or three-pence are generally well attended; but these, indeed, are seldom offered.

I would suggest that, in addition to these evening lectures (which, being delivered by clergymen, and in a district situate somewhat remotely from places of public worship, were, in the case referred to, perhaps very properly, limited exclusively to topics of religious instruction), discourses should also be delivered to the men in the winter evenings, on popular science, on the ordinary phenomena of the domestic and work-day life, in a familiar style, and in language devoid of technicalities, and adapted to the comprehension of the hearers. Thus a taste for the acquisition of knowledge would be created; this taste subsequently nourished and elevated; and the accursed beer-house, the publican's parlour, gradually forsaken. The poison would then have its antidote near at hand. Unquestionably a large amount of existing improvidence, folly, waste, sensuality, disease, nay, wickedness, in society, may justly be attributed to the deplorable ignorance which prevails, respecting the laws which regulate our being; which govern our *physical* welfare, and by which so large a portion of the *moral* universe is swayed. What most concerns the things which occupy his attention in his daily life, is, during his childhood and youth, sedulously kept from the knowledge of the working man.

Nor should our endeavours to infuse a little useful knowledge among the adult operative class be confined to the men. The women are even more ignorant; and, for obvious reasons, it is yet more important that *they* should be instructed in the duties of life; in the plain rules of conduct by which female life should be regulated. "All would be well, indeed," says Southwood Smith, "if the marriage ceremony, which converts the girl into a wife, conferred upon the wife the qualities which should be possessed by the mother." But it is very rare to find a young female capable of even the least trying part of early training. Yet a girl brought up in the ordinary mode, and in the domestic circle of her class, environed by all the corrupting agents on which I have dwelt, and with scarcely a redeeming or wholesome influence, is at the age of eighteen or nineteen entrusted with the direction and control of the earliest impressions that are made upon the human being; and the mighty and permanent physical, moral, and intellectual results, that arise out of those impressions! Female classes should be formed; to consist of the wives and daughters of colliers, and directed by such ladies of the neighbourhood as are willing and capable to aid in such a good work. These classes should teach—not the *accomplishments* of domestic life, not music or dancing, or frivolous crochet—but a fitness for the earnest work, the solemn duties of existence: cooking, the fabrication of children's clothes (which very few females of this class understand), domestic economy, how to reclaim an erring husband, how to secure and direct aright the affections of a child.

I have now sketched, in rapid outline, the most obvious and practicable modes of procedure, whereby the condition of a very large and interesting section of our labouring population may be materially improved, and their moral standard permanently raised. I have shown that there is much in the power of the masters to achieve. Let us hope that *they* will henceforth devote to the subject that serious attention which it merits at their hands. Their wealth-producers may surely claim their sympathies, their aid, direction, and co-operation, in whatever promises to benefit their condition, and promote their welfare. Moreover, the employers themselves, in a worldly point of view, are interested in the matter. Is a sickly and debased workman of the same value (as a wealth-producer) as one of healthy tone, both of mind and body? Assuredly not. But let us not pursue this line of argument. This is an age of sublime conflict; of the war of intelligence and philanthropy with all the foul protean forms of ignorance. Peaceful science has its victories as well as war. Its trophies are increase of rational enjoyment and happiness among the masses, healthy dwellings, a smiling infancy, a well-trained youth, a contented populace, a diminished mortality. The professors of the healing art, battling with disease and misery, waging eternal conflict against bodily and spiritual uncleanness; and enforcing, through good report and through evil report, the principles and practical application of sanitary reform, have already obtained, and shall continue to reap, more glorious conquests than red-handed Mars—victories over the direst enemies to man's advancement, the bitterest foes to human progress; compared with which, the fields of Marathon, of Bannockburn, of Waterloo, shall pale their splendour. What does Byron say?—

> "The drying of a single tear, has more
> Of honest fame than shedding seas of gore."

Hindley, near Wigan, 1856.

---

